# Impact of Oxidative Stress in Fetal Programming

**DOI:** 10.1155/2012/582748

**Published:** 2012-07-11

**Authors:** Loren P. Thompson, Yazan Al-Hasan

**Affiliations:** Department of Obstetrics, Gynecology and Reproductive Sciences, University of Maryland School of Medicine, 11-029 Bressler Research Building, 655 W. Baltimore Street, Baltimore, MD 21201, USA

## Abstract

Intrauterine stress induces increased risk of adult disease through fetal programming mechanisms. Oxidative stress can be generated by several conditions, such as, prenatal hypoxia, maternal under- and overnutrition, and excessive glucocorticoid exposure. The role of oxidant molecules as signaling factors in fetal programming via epigenetic mechanisms is discussed. By linking oxidative stress with dysregulation of specific target genes, we may be able to develop therapeutic strategies that protect against organ dysfunction in the programmed offspring.

## 1. Introduction

This paper addresses the emerging role of oxidant molecules in fetal programming. Oxidative stress is associated with the generation of reactive oxygen species (ROS), which have both physiologic and pathologic roles in the placenta [1], embryo [2], and the fetus [3–5]. We will address recent reviews and current studies that present evidence identifying the impact of oxidative stress on the fetus and how it may contribute to the permanent alterations in the offspring through programming mechanisms.

## 2. Intrauterine Stress and Fetal Programming

Epidemiological evidence and animal model studies have identified a clear association between low birth weight and an increased incidence of hypertension, type II diabetes, metabolic syndrome, insulin resistance, and obesity [6–9]. This was first reported by Dr. David Barker in relation to the inverse relationship between birth weight and mortality rate due to cardiovascular disease [10–12] and systolic blood pressure [13]. Subsequently, babies of higher than normal birth weight were shown to have increased risk of metabolic disorders as adults [14, 15]. Extensive study has lead to identifying additional factors that include altered nutrition, glucocorticoid exposure, and prenatal hypoxia as intrauterine stressors initiating fetal programming [8, 9].

The relationship between adult disease (e.g., hypertension, insulin resistance, diabetes) and birth weight forms a U-shape curve demonstrating increased risk with both low [10–12] and high birth weight [16, 17]. The impact of intrauterine stress on the affected offspring is influenced by the severity and duration of the insult, as well as, the gestational age of fetal exposure [8, 9]. In conditions of maternal undernutrition, the fetus adapts by reducing its metabolic energy supply to protect critical organs for survival, first described as the Thrifty Phenotype Hypothesis [18]. With further study, the Predictive Adaptive Hypothesis was proposed to describe the fetal response to an anticipated undernourished postnatal environment [19]. Overnutrition (i.e., high fat and carbohydrate diet) during pregnancy also imposes an intrauterine challenge because the fetus is unable to properly regulate its nutrient excess, resulting in a greater than normal birth weight [20]. This is likely due to an imbalance of the appropriate complement of nutrients required for proper development of the fetal organs [9, 21]. Thus, high or low birth weight, resulting from altered growth patterns during intrauterine stress, carries an increased risk of adult disease for the offspring [6–9].

## 3. Role of Oxidative Stress in Normal ****Fetal Development

The development of the embryo occurs in a relatively low-oxygen environment. It is highly sensitive to injury to oxidant molecules because of its low antioxidant capacity [22, 23]. As placentation progresses, there is increased oxygen transfer, which increases the cellular generation of ROS [1]. This initiates a switch in the cellular redox state, from reduced to oxidized, acting as a driving force for cell differentiation [23, 24]. ROS serve as signaling molecules that induce transcription of several genes (e.g. *HIF1A, CREB1, NFKB1*) important in oxygen sensing, cell differentiation, and proliferation [1, 24, 25]. With placental maturation, there is increased oxygen transfer to the developing fetus, which is required for sustaining the increased metabolic rate during the rapid fetal growth phase [2].While both nutrition and oxygenation are important for fetal growth, identifying the specific effect of hypoxia in the absence of altered nutrition is important for understanding the role of hypoxia in fetal programming. Recent study has identified the effect of reduced oxygenation, independent of nutrition, using a chick embryo model [26]. Incubation of fertilized eggs from hens at high-altitude resulted in reduced fetal growth compared to eggs obtained from hens at sea level. The restricted fetal growth occurring at high-altitude was corrected by incubating fertilized eggs from high-altitude hens at sea level or by oxygen supplementation that matched sea level conditions. Thus, in the presence of adequate nutrition, reduced oxygenation can negatively impact normal growth and development.

Recent animal studies have identified an important role of oxidant molecules in fetal cardiovascular function under normal conditions [3–5]. Antioxidant administration of melatonin or vitamin C to pregnant sheep increased umbilical vascular [5] and fetal femoral artery conductance [4], respectively, by mechanisms associated with increased nitric oxide (NO) bioavailability following decreased superoxide anion levels. In addition, inhibition of superoxide anion generation by allopurinol, a xanthine oxidase inhibitor, attenuated the fetal sheep pressor responses to phenylephrine and enhanced umbilical vascular conductance by altering NO bioavailability and activation of *β*1 adrenoceptor mechanisms, respectively [3]. Thus, oxidant molecules play an important physiological role in normal fetal cardiovascular homeostasis. A delicate balance may exist between optimal oxygen levels and the generation of ROS molecules during gestational periods of embryonic and fetal development.

## 4. Intrauterine Stress and Hypoxia

Fetal hypoxia is one of the most significant challenges facing obstetric clinical practice. Intrauterine stress can be generated under conditions of placental insufficiency [27], preeclampsia [28], high-altitude environments [29–33], and exposure to toxic substances [34]. High-altitude living exposes populations to hypobaric hypoxia and imposes a significant challenge to the pregnant woman and her developing fetus. Low birth weight and preeclampsia are common characteristics of high-altitude pregnancies [29–33]. The risk of small size at birth is associated with the increased incidence of high blood pressure and heart disease in the adult offspring [31]. While preeclampsia is associated with oxidative stress [28], it is unclear whether oxidative stress contributes to pregnancy complications that occur at high-altitude.

 Animal studies have shown that intrauterine hypoxia induces fetal growth restriction [26, 35], cardiovascular dysfunction [36, 37], and multiorgan morbidities of the fetus associated with brain [38–40], heart [41–44], liver [45–47], and kidney [7, 48]. Further, recent studies have linked prenatal hypoxia to an increased risk of cardiac [4, 5, 36, 46, 49, 50], vascular [51–53], and metabolic [46] dysfunction in the offspring. Thus, the condition of intrauterine hypoxia impacts the fetus on a multiorgan level and increases the risk of adult disease via mechanisms associated with fetal programming [8, 54].

Understanding the impact of intrauterine hypoxia and its generation of ROS during gestation is important for understanding the consequences of both fetal and neonatal outcomes. The effect of ROS on both genetic and epigenetic mechanisms is implicated in the underlying cause of fetal programming although further study is needed. Understanding the contribution of ROS in fetal programming will greatly improve our understanding of the impact of oxidative stress on the long-term consequences contributing to organ injury of programmed offspring.

## 5. Generation of Oxidative Stress

Oxidative stress occurs when the generation of reactive oxidant molecules exceeds the capacity of the cell's antioxidant defense mechanisms. Both enzymatic (i.e., MnSOD, CuZnSOD, Catalase) and nonenzymatic (GSH/GSSG, peroxiredoxin, thioredoxin, ascorbic acid or Vitamin C, tocopherol or Vitamin E) antioxidant pathways are present to combat excessive ROS generation. The major ROS molecules include superoxide anion (O_2_^−^), hydroxyl radical (^•^OH), and hydrogen peroxide (H_2_O_2_), as well as, others generated by their interaction with reactive molecules, such as, NO and peroxynitrite [55]. Superoxide anions are generated by plasmalemmal- and mitochondrial-associated NADPH oxidases, xanthine oxidase, mitochondrial respiration and by products of several metabolic pathways, such as, fatty acid oxidation [55]. The predominant generators of ROS are cell type specific and their formation is dependent on the cell's metabolic energetics [56, 57]. Mitochondria are considered primary generators of O_2_^−^ under conditions of reduced oxygen [55, 56] although xanthine oxidase has been shown to contribute significantly to cardiovascular homeostasis in fetal sheep [3–5]. Under normal oxygen conditions, the electron transport chain leaks O_2_^−^ from Complexes I and III, which are normally balanced by mitochondrial SOD (i.e., MnSOD) [57]. With hypoxia, a reduction of molecular O_2_, as the electron acceptor at terminal complex IV, can result in leakage of electrons and generation of O_2_^−^ leading to oxidative stress [57].

Targets of oxidative stress include phospholipid membranes, proteins, and nucleic acids [58]. Plasma and organelle membranes are particularly vulnerable to oxidative stress because phospholipids are easily oxidized by O_2_^−^. ROS molecules can directly interact with both proteins and DNA to oxidize aminoacids, which disrupt normal structure and function. In addition, mitochondria are predominant targets of oxidative stress because the mitochondrial genome lacks histones and has fewer DNA repair mechanisms than the nucleus [59]. Damage to mitochondrial proteins and membranes can lead to further mitochondrial dysfunction. This can result in a positive feedback mechanism by which failure of only a few mitochondria, injured by oxidative stress, can recruit an entire network of mitochondria to fail [60]. Thus, organs that have a high reliance on oxidative phosphorylation and *β*-fatty acid oxidation may be more vulnerable to oxidative stress both *in utero* and after birth.

## 6. Role of Reactive Oxygen Molecules ****in Epigenesis

Oxidant molecules can directly interact with DNA base pairs causing both genetic, as well as, epigenetic changes, the latter through alterations in DNA methylation and histone modification [61]. The cellular redox status influences gene expression and cell differentiation [23, 61]. The influence of ROS on epigenetic alterations in DNA methylation has been extensively studied in cancer [59]. Recently, the influence of ROS on DNA methylation is considered an important process of altered gene expression [59, 61]. Epigenesis is a process by which gene expression is either suppressed or enhanced without changes in primary DNA sequences but rather changes in the capacity of transcriptional control regions to induce gene expression [59].

DNA methylation is a prominent modification that leads to gene suppression of mRNA transcription and protein synthesis [59]. DNA methyltransferases methylate CpG islands, which influence the binding of transcription factors and/or their coregulators. Besides serving as signaling factors in transcription, ROS can interact directly with DNA resulting in oxidative damage and DNA breaks. Further, oxidant-mediated DNA breaks provide access to sites for DNA methyltransferases, which promote DNA methylation.

Modification of gene expression can also occur by altering the regulatory roles of histones. Chromatin is DNA wound around histones and gene expression is regulated by allowing access of transcription factors and RNA polymerase to DNA binding sites. Aminoterminal tails of histones are susceptible to posttranslational modification, such as, methylation, acetylation, phosphorylation, and ubiquitination [59]. Besides acting as signaling molecules in modifying histone function, ROS can also directly interact with histones resulting in disruption of normal gene expression.

## 7. Oxidative Stress and Fetal Programming

Fetal origins of adult disease are associated with several causative mechanisms depending on the conditions of intrauterine stress [6, 54, 62]. The role of oxidative stress in fetal programming is supported by epidemiological evidence of oxidant indices and low birth weight in association with type 2 diabetes [63], cardiovascular disease [64], and preeclampsia [28]. Thus, oxidative stress may be a connecting link between intrauterine insult and programming consequences after birth.

Children (8–13 years old) born growth restricted exhibit increased levels of lipid peroxidation and have higher blood pressures compared to age-matched children of normal birth weight [65–67]. Intrauterine growth-restricted (IUGR) infants had increased serum levels of both oxidant (lipid peroxidation and DNA damage) and antioxidant indices (superoxide dismutase, catalase, glutathione peroxidase) [68]. Indices of lipid peroxidation were elevated in placenta of women with preeclampsia [69, 70] and in plasma or serum of women with IUGR fetuses [71–73]. Oxidative stress associated with pregnancy complications may be a contributing factor in postnatal consequences of the neonate.

Animal studies support the hypothesis that oxidative stress induces programmed phenotypes in the adult offspring. Pregnant rats fed a low-protein diet have offspring (10–12 weeks old) that exhibit elevated arterial blood pressures and increased vasoconstrictor responsiveness to angiotensin II [74]. Prenatal treatment with the antioxidant agent, lazaroid, prevented the alterations in the cardiovascular responses associated with maternal protein restriction. In a separate study, maternal low-protein diet impaired the recovery to ischemia/reperfusion injury of offspring rat hearts, which was reduced with postnatal administration of the antioxidant, N-acetylcysteine [75]. Maternal administration of the steroid, dexamethasone, generates offspring that exhibit increased ROS production (i.e., H_2_O_2_) in the coronary circulation [76] and in mitochondria of hearts of programmed sheep [77]. Postnatal glucocorticoid administration to 1–6-day-old rat pups increased vasoconstrictor responsiveness to KCl and the thromboxane analog, U46619, which was restored to normal function with Vitamin C and E treatment [78] suggesting that excessive glucocorticoid exposure promotes oxidative stress.

Prenatal hypoxia has been shown to generate oxidative stress in fetal hearts in a variety of animal species, such as, sheep [79], rat [36, 41], and guinea pig [42–44]. Maternal hypoxia increases expression levels of fetal cardiac inducible NO synthase [42, 44], nitrotyrosine [36, 43], heat shock protein 70 (HSP70) [36, 80], proinflammatory cytokines [81], and matrix metalloproteinases [44, 81–83] suggesting the generation of oxidative and inflammatory stress as causative. Prenatal treatment with the antioxidant, N-acetylcysteine, inhibited the hypoxia-induced increase in peroxynitrite levels and fibrosis in fetal guinea pig heart ventricles [43]. Prenatal vitamin C inhibited the hypoxia-induced increase in fetal cardiac HSP70 expression and adult myocardial contractility associated with *β*1 adrenoceptor stimulation in rat offspring [36]. With prenatal hypoxia, hearts of 6 month old male rat offspring exhibit reduced recovery to ischemia/reperfusion injury [80] in a sex-dependent manner with males more vulnerable than females [84] suggesting a protective influence of estrogen. In addition, exposure to prenatal hypoxia reduces cardiac efficiency of isolated perfused hearts of 4- and 12-month-old rat offspring during reperfusion following an ischemic period. The prenatal hypoxic insult causes growth restriction of the offspring and alters cardiac performance attributed to reduced glucose oxidation [49]. Furthermore, hyperoxic exposure of neonatal rats increases ROS production and is associated with increased systolic blood pressure and vascular dysfunction in adults [85]. Taken together, these studies suggest that oxidative stress is an important stressor that impacts the fetal heart and initiates cardiovascular programming of the offspring.

Prenatal hypoxia has also been shown to alter vascular reactivity of the adult offspring [51–53]. Myogenic response of mesenteric arteries from male 7-month-old rats exposed to prenatal hypoxia was greater compared to their normoxic controls [53]. This was attributed to reduced NO and vasodilator prostaglandin bioavailability in the offspring arteries. In separate studies, isolated mesenteric arteries of 4- and 12-month old rats exposed to hypoxia *in utero* exhibited decreased flow-mediated vasodilation in a pressure myograph [51] and endothelium-dependent relaxation to methacholine stimulation in a wire myograph [52]. A hypoxia-induced decrease in dilator responsiveness was associated with a decrease in NO-dependent relaxation in the offspring that was attributed to mechanisms associated with a premature aging process. The role of oxidative stress was not investigated.

In addition, other organs are affected by oxidative stress during fetal development. Reduced placental perfusion is associated with oxidative stress in IUGR fetuses [72, 86–89]. Pancreatic *β* cells of offspring of IUGR fetuses are vulnerable to oxidative stress because of a low-antioxidant capacity and exhibit increased ROS production and impaired ATP generation [90]. Prenatal hypoxia increased DNA fragmentation and lipid peroxidation in fetal guinea pig livers, which was reversed by the antioxidant, N-acetylcysteine [46]. In livers of IUGR offspring, there is an upregulation of MnSOD as a compensatory adaptation that counters the decrease in pyruvate oxidation [91]. Prenatal hypoxia decreases insulin-signaling proteins in livers but not skeletal muscle of rat offspring [47]. Oxidative stress in hepatic tissue renders the adult liver susceptible to nonalcohol-associated fatty liver disease [92]. The kidney also plays an important role in programmed hypertension when exposed to an intrauterine environment that alters oxidative stress pathways. A homozygous NO synthase 3 deficient mouse was cross bred to generate paternally and maternally derived heterozygous offspring with high blood pressures [93]. Offspring that developed in a high-oxidative stress environment exhibited increased expression of peroxiredoxin, HSPB6, SOD-1, and PPAR*γ* in kidney but not liver tissue, compared to their counterparts who developed in a normal environment, identifying a role of oxidative stress in contributing to developmental programming of kidney-associated hypertension. Overall, reduced fetal oxygenation may impart organ-specific dysfunction via oxidative stress.

## 8. Oxidative Stress and Epigenetic Mechanisms of Fetal Programming

Animal studies have generated strong support for the role of epigenesis in response to altered maternal nutrition as an important programming mechanism in the offspring [94–96]. Both over- and undernutrition have been reported to induce DNA methylation of selected gene promoter regions [94–96]. Oxidative stress has also been identified as a contributing factor in epigenetic mechanisms [23, 61, 95, 96]. The role of oxidative stress in contributing to epigenetic modifications, independent of nutritional effects, warrants further study. The challenge is to link oxidative stress during pregnancy to specific target sites of epigenetic change in the offspring. Because ROS interacts promiscuously, we are limited in our ability to identify ROS-specific signaling within the cell [97].

Despite these limitations, intrauterine hypoxia and IUGR have been shown to alter DNA methylation of selected genes in several tissues including placenta [98], heart [99, 100], pancreas [101], liver [95], adrenal gland [102] and pulmonary arteries [103]. The following studies have identified specific target genes in fetal and offspring organs whose expression is altered by changes in DNA methylation associated with intrauterine hypoxia, IUGR, and protein restriction.

Chronic fetal hypoxia has been shown to decrease protein kinase C epsilon (PKC*ɛ*) mRNA and protein expression in fetal and adult rat hearts [84]. The hypoxia-induced decrease in PKC*ɛ* expression is associated with methylation of CpG sites, specific for two SP1 binding sites in the PKC*ɛ* promoter region in both *ex vivo* treatment of fetal hearts and in H9c2 cells, an isolated embryonic ventricular myocyte cell line [99]. The epigenetic modification by hypoxia was further demonstrated by restoring the decrease in PKC*ɛ* mRNA and protein expression by inhibiting DNA methyltransferase I with 5-aza-2′-deoxycytodine. This study links the epigenetic modification of PKC*ɛ* expression in adult offspring hearts with prenatal exposure to chronic hypoxia.

In pulmonary arteries of offspring mice exposed to two weeks of hypoxia during pregnancy, DNA methylation was increased and was associated with reduced pulmonary artery endothelium-dependent vasodilation [103]. The hypoxia-induced decrease in dilator responses to acetylcholine was reversed in the presence of histone deacetylase inhibitors, buryrate, and trichostatin A, in association with reduced DNA methylation.

The fetal pancreas is vulnerable to oxidative stress because of its reduced antioxidant capacity [104, 105]. In IUGR, rat pancreas exhibited reduced gene expression of the transcription factor, PDX1, which regulates pancreatic development and *β*-cell differentiation [101]. Pancreatic *β* cells of IUGR rat fetuses, exhibited reduced expression of *Pdx1*, a homeobox gene required for pancreatic development and insulin production, as a result of epigenetic modifications throughout development [106]. The inhibition of *Pdx1* expression progressed in IUGR fetuses after the recruitment of histone deacetylase 1 to the *Pdx1* promoter and subsequent demethylation of histones H3 and H4. In 2-week-old and 6-month old islets [106], H3 was permanently methylated. It is proposed that progressive DNA methylation inhibits gene expression and may link gene silencing in the *β* cells to the development of a diabetic phenotype [84]. Although not hypoxia-induced, protein restriction increases the expression of peroxisome-proliferator-activated receptor alpha (PPAR*α*) protein and glucocorticoid receptors in the offspring rat liver and is associated with hypomethylation of their promoter regions [96]. In adrenal gland of rat offspring (4-week-old), maternal protein restriction increased expression of angiotensin receptor AT1b, which correlated with hypomethylation of its promoter and was reversed with maternal administration of 11*β*-hydroxylase inhibitor, metyrapone, suggesting that maternal glucocorticoid induces changes in methylation in fetal adrenal glands [102].

Further, excess glucocorticoid exposure during pregnancy was also shown to induce epigenetic responses contributing to programming effects in the offspring [107]. For example, glucocorticoid exposure of isolated cultured rat hepatocytes induced DNA demethylation of a specific gene locus (*Tat*) associated with a glucocorticoid response unit [108]. This demonstrated that glucocorticoid may regulate DNA demethylation as a mechanism of modulating gene expression to subsequent stimulation by glucocorticoid receptors. In embryonic cortical neural stem cells, glucocorticoid decreased global DNA methylation, which is retained in daughter cells [109]. These studies implicate glucocorticoid as activating sustained epigenetic modifications that may result in long-term consequences associated with specific cell-related phenotypes.

## 9. Conclusions

ROS generated by a variety of intrauterine conditions may be one of the key downstream mediators that initiates epigenesis and programming of the offspring. ROS generation normally is balanced by the cell's antioxidant defense mechanisms, which maintains its redox state and is important in physiological regulation in both the embryo and fetus. Prenatal hypoxia, nutritional deficiency/excess, and glucocorticoid exposure are each capable of generating excessive ROS levels by differing mechanisms. Organ-specific responses are dependent on the relative balance between ROS generation and the antioxidant capacity of the cell. Identifying the impact of oxidative stress on gene targets will be important for understanding the cell-specific responses to intrauterine stress, as well as, developing therapeutic strategies for alleviating long-term programmed consequences associated with adult disease.
